# Prenatal Identification of a Missense Mutation of the L1CAM Gene Associated With Hydrocephalus Using Next-Generation Sequencing

**DOI:** 10.7759/cureus.55142

**Published:** 2024-02-28

**Authors:** Sotirios Sotiriou, Athina A Samara, Eleftherios Anastasakis, Athanasios Zikopoulos, Ioannis Papoulidis, Emmanouil Manolakos, Efterpi Pavlidou, Chara Skentou

**Affiliations:** 1 Department of Embryology, University of Thessaly, Larissa, GRC; 2 Clinical Laboratory Genetics, Access To Genome P.C., Athens, GRC; 3 Department of Speech and Language Therapy, University of Ioannina, Ioannina, GRC; 4 Department of Obstetrics and Gynecology, University of Thessaly, Larissa, GRC

**Keywords:** next-generation sequencing (ngs), corpus callosum (cc) agenesia, iugr, hydrocephalus, l1cam gene

## Abstract

We present the case of a 35-year-old pregnant woman who visited our department for a routine ultrasonography screening scan for fetus anatomy during the 22nd week of gestation. Our report revealed a male fetus with marked hydrocephalus and severe intrauterine growth retardation. After extensive counseling, the couple decided to proceed with an invasive diagnosis via amniocentesis. The cytogenetic analysis showed findings related to clinical history and ultrasound findings related to the presence of a nucleotide change in c.578T>C with an amino acid change in p.Leu198Pro of the *L1CAM *gene. The result was reported as a hemizygote missense *L1CAM *gene variant of unknown significance. After extensive parental counseling, the couple decided on pregnancy termination. We report the present case of *L1CAM *mutation in p.Leu198Pro to add to the limited knowledge regarding the clinical presentation of mutations of the *L1CAM *gene with emphasis on prenatal diagnosis.

## Introduction

Mutations of the L1 cell adhesion molecule (*L1CAM*) gene can be detected in approximately 1 in 30,000 male births. *L1CAM *mutations are associated with clinical features of partial agenesis of the corpus callosum [1-4], hydrocephalus due to aqueductal stenosis [5-9], hydrocephalus with Hirschsprung’s disease [10], as well as MASA (mental retardation, aphasia, shuffling gait, and adducted thumbs) [11-13] and CRASH (corpus callosum hypoplasia, retardation, adducted thumbs, spastic paraparesis, and hydrocephalus) [14] syndromes. The *L1CAM *gene follows a sex-linked mode of inheritance, and the clinical manifestations usually involve the male gender [15-20]. Μutations of the *L1CAM *gene are rare chromosomal aberrations [21-23].

Most cases are described with the term L1 syndrome, including a group of X-linked recessive disorders with a common genetic basis and clinical presentation ranging from mild to severe [24]. Spastic paraplegia, MASA syndrome, X-linked complicated corpus callosum dysgenesis, and X-linked hydrocephalus with stenosis of the aqueduct of Sylvius are common disorders of L1 syndrome [1]. In the current literature, L1 syndrome can be found as L1CAM syndrome, named after the causative gene, or as CRASH syndrome, an acronym for its primary clinical features, i.e., corpus callosum hypoplasia, intellectual disability, adducted thumbs, spasticity, and hydrocephalus [2-4]. The clinical manifestation of L1CAM differs from intellectual disability to aphasia and spasticity. A diagnosis can be made with a genetic test after birth or prenatally through an invasive procedure such as amniocentesis or chorionic villus sampling [6,7].

Most cases arise from different variants in the *L1CAM *gene [24]. The *L1CAM *gene consists of 29 exons and the producing protein, a transmembrane neuron surface glycoprotein that has 1,257 amino acids and is responsible for the creation of neural synapses [25]. This protein is essential for correct brain structural development and, by extension, is necessary for proper brain function. To date, 169 different *L1CAM *mutations have been reported [24].

Here, we report an interesting case of an *L1CAM *mutation in p.Leu198Pro in a male fetus with marked hydrocephalus, suspected partial agenesis of the corpus callosum, and severe intrauterine growth retardation (IUGR). The present report aims to add to the limited knowledge regarding the clinical presentation of mutations of the *L1CAM *gene with an emphasis on the importance of prenatal diagnosis.

## Case presentation

**Figure 1:**
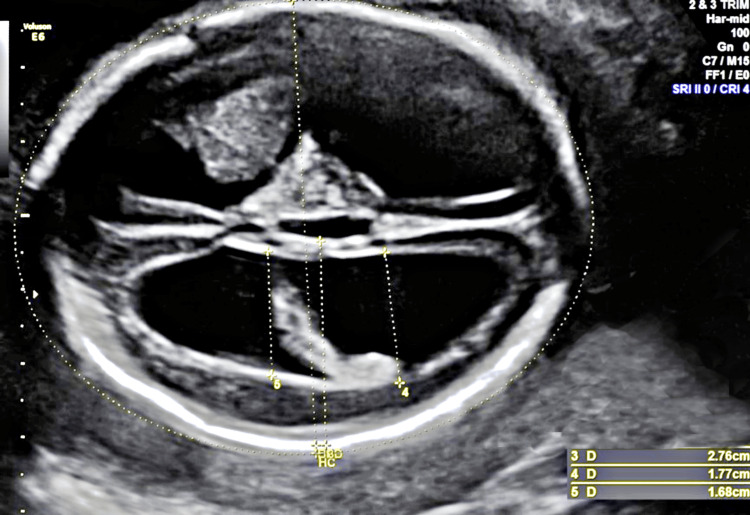
Ultrasound of the fetal head depicting ventriculomegaly.

A 35-year-old pregnant woman (gravida 2, para 1) visited the prenatal ultrasound department of our hospital for a routine ultrasonography screening scan for fetus anatomy during the 22nd week of gestation. The woman had experienced an uneventful pregnancy up to that point. Moreover, the maternal and paternal medical and family history were unremarkable. The ultrasound examination revealed a male fetus with marked hydrocephalus, suspected partial agenesis of the corpus callosum, and severe IUGR. The measurements of the anterior and posterior ventricles were 13.5 mm and 17.7 mm, respectively (Figure 1).

**Figure 2:**
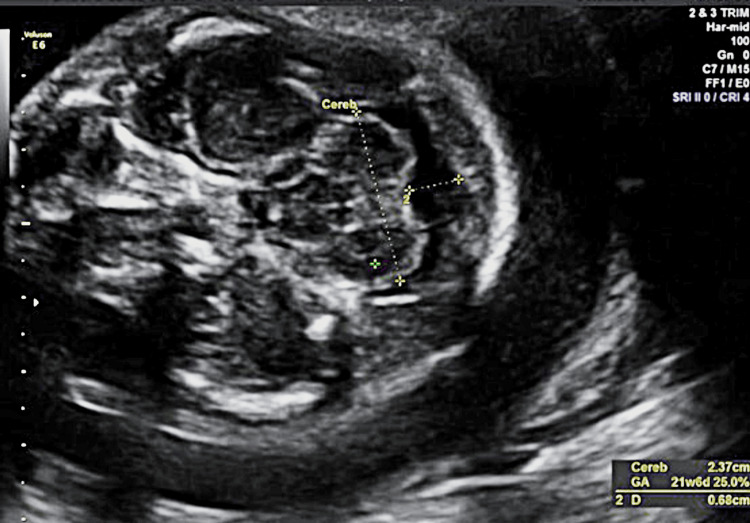
Fetal head images and cerebral measurements.

Figure 2 depicts other findings related to the fetal head. More specifically, other possible congenital anatomical hydrocephalus causes were not identified based on the normal posterior brain fossa and fetal spine ultrasound appearance. Furthermore, other anatomical abnormalities were not identified. The estimated fetal weight was below the fifth centile for the specific week of pregnancy based on biparietal diameter, abdominal circumference, and femur length measurements, and the diagnosis of IUGR was established.

After extensive counseling for possible hydrocephalus causes, the couple decided to proceed with an invasive diagnosis via amniocentesis, and a whole DNA sample was isolated. Amplification of the under-examined regions was performed using the Ion AmpliSeq Exome RDY kit, followed by nucleotide sequencing using the Ion Chef Instrument in combination with the Ion GeneStudio S5 System.

Whole exosome sequencing analysis was performed on 4,432 genes that are known to be associated with genetic diseases, and syndromes with clinical significance were analyzed. The genes analyzed were based on the American College of Medical Genetics and Genomics (ACMG) consensus. The evaluation and interpretation of the data were based on the clinical phenotype. Analysis was performed using bioinformatics analysis systems Alamut Visual and VarSome Clinical (Saphetor) a CE IVD-certified and HIPAA-compliant platform. All findings resulting from the above analysis were evaluated based on the international literature and the guidelines of the ACMG [26].

The criteria for evaluating and reporting findings include nucleotide changes that are evaluated with the currently available data as non-pathogenic or possibly non-pathogenic. These are not reported, whereas pathogenic or possibly pathogenic mutations are related to the clinical criteria of the referral case.

**Figure 3:**
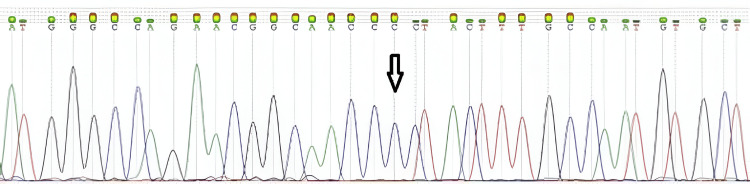
Sanger sequencing results of the fetus showing the presence of the hemizygous c.578T>C mutation in the L1CAM gene.

The cytogenetic analysis showed a 46, XY karyotype. Findings related to the clinical history and ultrasound were related to the presence of a nucleotide change in c.578T>C with an amino acid change in p.Leu198Pro of the *L1CAM *gene. The result was reported as a hemizygote missense *L1CAM* gene variant of unknown significance (Figure 3).

**Figure 4:**
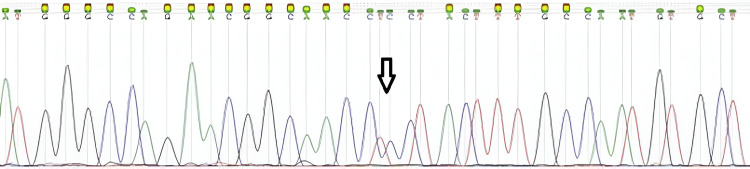
Sanger sequencing results of the mother showing the presence of the heterozygous c.578T>C mutation in the L1CAM gene.

Maternal testing was recommended to clarify whether the nucleotide change of p.Leu198Pro had been inherited from the mother or whether it had arisen de novo. Sanger sequencing of the gene segment containing the specific nucleotide change of p.Leu198Pro was performed using maternal blood (Figure 4).

Results revealed that the mother carried the specific change in heterozygosity, indicating its maternal origin in the fetus. Screening of other potentially affected male family members was also recommended. After extensive parental counseling, the couple decided on pregnancy termination.

## Discussion

The severity of symptoms and functional impairment of a patient with L1 syndrome is directly related to the type and severity of the *L1CAM *variant causing the disorder [6,7]. There is no cure for L1 syndrome, and the prognosis is often poor [8,9]. Life expectancy for people with L1 syndrome depends on the clinical severity of the disease, varying dramatically from dying shortly after birth to reaching adulthood [2]. Treatment strategies mainly consist of supportive care, which aims to improve the quality of life of these patients and minimize functional impairment [3,10].

The L1 cell adhesion molecule, a member of the immunoglobulin adhesion protein superfamily, is composed of six immunoglobulin-like domains and fibronectin type III-like repeats in the extracellular region and a short cytoplasmic tail [27,28]. Most reported mutations include missense, nonsense, small insertions, or deletions and splice-site alterations that are distributed throughout the large extracellular domain of the *L1* gene. The nonsense and frameshift variants lead to truncation of the L1 protein. Missense mutations account for over one-third of pathological *L1* mutations described [29]. Our case provides a novel insight because the nucleotide change of c.578T>C with an amino acid change of p.Leu198Pro to the *L1CAM *gene has not been reported, so our findings on the disease spectrum linked to this specific mutation and its prenatal diagnosis are new. Using next-generation sequencing analysis, this study revealed a new hemizygote missense *L1CAM *gene mutation, thus expanding the disease spectrum of L1 syndrome. The clinical importance of this mutation needs to be verified by analyzing a larger number of samples.

Findings that cannot be interpreted because they have not been reported to date in the international literature, i.e., variants of unknown significance, only refer to genes related to the possible specific indications of the reference case. Regarding the clinical interpretation of our case, based on the cytogenetic analysis results, the nucleotide change to c.578T>C with an amino acid change to p.Leu198Pro of the *L1CAM* gene has not been reported in the ClinVar and Decipher databases nor has it been reported until now in the international literature. According to the ACMG/AMP guidelines [26], the amino acid change in p.Leu198Pro is listed as a gene mutation of unknown significance (Table 1).

**Table 1:** Standards and guidelines for the interpretation of the p.Leu193Pro variant.

Standards and guidelines
PM1 Pathogenic Moderate: UniProt protein L1CAM_HUMAN domain ‘Ig-like C2-type 2’ has 15 non-VUS missense/in-frame/non-synonymous, variants (15 pathogenic and 0 benign), pathogenicity = 100.0% which is more than threshold 50.0%
PM1 Pathogenic Moderate: Variant not found in gnomAD exomes (good gnomAD exomes coverage = 87.1). Variant not found in gnomAD genomes (good gnomAD genomes coverage = 22.1)
PP3 Pathogenic Supporting: Pathogenic computational verdict based on pathogenic predictions from BayesDel_addAF, CADD, DANN, DEOGEN2, FATHMM-MKL, MCAP, MVP, MutationAssessor, MutationTaster, Polyphen2-HVAR, PrimateAI, REVEL and SIFT vs 1 benign prediction from LIST-S2

In the present report, we describe the first prenatally diagnosed case of a missense mutation of the *L1CAM *gene associated with hydrocephalus, IUGR, and possible partial agenesis of the corpus callosum using next-generation sequencing. This missense mutation of the *L1CAM *gene, as indicated by our findings, appears to be involved in abnormal brain clinical features contributing to the expansion of the L1 syndrome spectrum associated with these pathologies.

## Conclusions

The L1 syndrome should be considered in male fetuses with hydrocephalus and abnormal brain ultrasound findings related to an abnormal corpus callosum appearance when other possible ventriculomegaly causes have been excluded. Our case suggests that pathogenic missense mutations affecting key amino acid residues are most likely to lead to a severe phenotype based on the severity of the ultrasound features. Additional studies are needed to investigate the pathogenicity of this mutation and its definite association with fetal hydrocephalus. We report the present case of *L1CAM *mutation in p.Leu198Pro to add to the limited knowledge about the clinical presentation of mutations of the *L1CAM *gene with emphasis on prenatal diagnosis.
